# A case of stiff dog syndrome associated with anti-glutamic acid decarboxylase antibodies

**DOI:** 10.1186/s40734-017-0053-3

**Published:** 2017-05-10

**Authors:** Theresa E. Pancotto, John H. Rossmeisl

**Affiliations:** 1 Department of Small Animal Clinical Sciences (Pancotto, Rossmeisl), Virginia-Maryland College of Veterinary Medicine, Virginia Tech, Mail Code 0442, Blacksburg, VA 24061 USA; 2 Brain Tumor Center of Excellence, Comprehensive Cancer Center (Rossmeisl), Wake Forest University School of Medicine, Winston-Salem, NC USA

**Keywords:** Canine, Autoimmune, GAD antibodies, Stiff person syndrome

## Abstract

**Background:**

The stiff person syndrome (SPS) is a rare and debilitating autoimmune disorder with an unknown pathogenesis and variable clinical presentation that can present a diagnostic challenge. Although entities that clinically mimic stiff-person spectrum disorders (SPSD) have manifested in horses, they have not been reported in dogs.

**Case presentation:**

We describe a 2-year-old beagle dog presented for progressive attacks of muscular rigidity and lordosis with superimposed spasms of the appendicular muscles triggered by tactile stimulation which resulted in marked gait impairment. Resting electromyography revealed continuous motor unit activity in the axial musculature. Compared to age-matched healthy beagle dogs, this patient had elevated glutamic acid decarboxylase antibody concentrations in serum and cerebrospinal fluid.

**Conclusion:**

This dog presented with phenotypic, electrodiagnostic, and immunologic criterion consistent with an SPSD, including elevated anti-GAD antibody titers, which we have termed the “stiff dog syndrome (SDS)”. Durable clinical improvement was achieved with symptomatic and immunosuppressive treatments including baclofen, gabapentin, prednisone, and intravenous immunoglobulin.

**Electronic supplementary material:**

The online version of this article (doi:10.1186/s40734-017-0053-3) contains supplementary material, which is available to authorized users.

## Background

In 1956 the “stiff man” syndrome was initially described in 14 patients with progressive neck and back stiffness, and episodic and painful muscular spasms resulting in impaired ambulation [1]. As subsequent studies have demonstrated that the syndrome affects twice as many women as men, the condition is now commonly referred to as the gender neutral stiff person syndrome (SPS) [2–4].

SPS is a rare and presumably immune mediated CNS disorder, although the pathophysiology remains unclear [4, 5]. The clinical features associated with classical SPS include: rigidity of the axial muscles, especially the abdominal and paraspinal musculature, leading to hyperlordosis which can progress to affect the limb musculature; episodic painful reciprocal spasms of agonist and antagonist muscles triggered by stress, auditory, or tactile stimuli; electromyographic evidence of continuous motor unit activity at rest, and the presence of glutamic acid decarboxylase (anti-GAD) antibodies [6]. SPS is now recognized as a heterogeneous group of autoimmune disorders (stiff person spectrum disorder [SPSD]) that are classified based on clinical presentation into classic SPS, paraneoplastic SPS, and SPS variants (focal or segmental-SPS, jerking-SPS, SPS-plus, and progressive encephalomyelitis with rigidity and myoclonus [PERM]) [3–6]. While anti-GAD antibodies are present in the majority of SPS patients, SPSD can be associated with antibodies against amphiphysin, gephyrin, glycine α-1 receptor, or gamma-aminobutyric acid receptor associated protein [5, 6]. Patients with SPSD frequently present with other autoimmune diseases including insulin dependent diabetes mellitus or Hashimoto’s thyroiditis [5].

Several spontaneous movement disorders described in dogs display similarities to their human analogs, including orthostatic tremor and some paroxysmal non-kinesigenic dyskinesias, which has promoted the use of dogs as models for human disease [7, 8]. Although a SPS-like condition has been described in horses with anti-GAD antibodies, to our knowledge this syndrome has not been reported in dogs [9, 10]. Here we describe a dog with a movement disorder meeting phenotypic, electrodiagnostic, and immunologic criterion of an SPSD, including serological evidence of anti-GAD antibodies, which was identified concurrently with underlying syringomyelia. This case illustrates the existence of a condition, which we refer to as the “stiff dog syndrome (SDS)”, similar to SPS in humans, and emphasizes the heterogeneous clinical manifestations of SPSD variants and the diagnostic challenges associated with this condition. Durable clinical improvement was achieved with symptomatic and immunosuppressive treatments.

## Case presentation

A 2-year-old spayed, female, beagle dog was presented to the Virginia Maryland College of Veterinary Medicine (VMCVM) Veterinary Teaching Hospital for progressive paroxysmal episodes of muscular hypertonicity, lordosis, and difficulty ambulating. Approximately three weeks prior to presentation to VMCVM, the patient starting exhibiting intermittent episodes of rigidity in all four limbs, each lasting <10 s, followed by a rapid return to normalcy. The events became longer and more frequent, culminating in multiple daily episodes of stiffness that interfered with ambulation. The patient was admitted to the referring veterinarian’s hospital (Day 0), and treated with intravenous diazepam (0.5 mg/kg/h constant rate infusion [CRI]), phenobarbital (4 mg/kg q 12 h), and methocarbamol (33 mg/kg q 8 h) which had no demonstrable effect on the frequency or severity of the episodes.

**Fig. 1:**
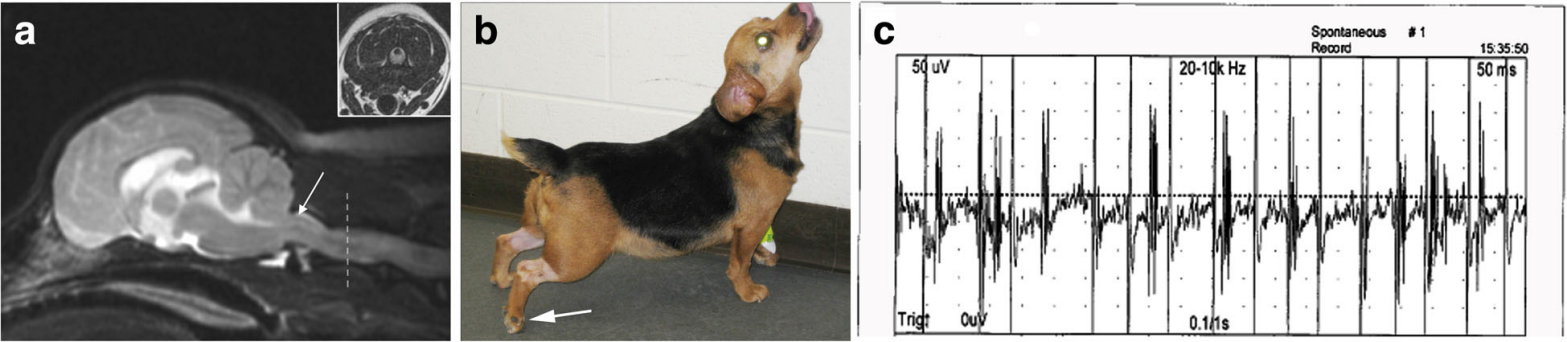
**a** Sagittal T2-weighted MR image revealing caudal occipital malformation, kinking of the caudal medulla, foramen magnum herniation (*arrow*), and a T2-hyperintense intramedullary signal within the spinal cord extending from C1 to C3. Transverse FLAIR image at the level of C2 (*broken arrow, inset*), demonstrating the hypointense syrinomyelic cavity in the dorsal spinal cord. **b** Lordotic posture resulting from axial muscular rigidity. Superimposed muscle spasms cause partial flexion of the limb joints, including the digits (*arrow*). **c** Resting EMG revealing continuous motor unit activity in the lumbar epaxial musculature

The following day (Day 1), an MRI examination of the brain was performed under general anesthesia, which revealed structural changes consistent with Chiari-like malformation (CM) and secondary syringomyelia (Fig. 1a). Cerebrospinal fluid (CSF) was collected from the cerebellomedullary cistern. The CSF protein concentration was elevated at 100 mg/dl (Table 1; reference interval <25 mg/dl) and a marked mononuclear pleocytosis was present with 981 total nucleated cells/μl (reference interval <3 cells/μl; differential 95.6% monocytes, 4.4% lymphocytes). The dog was empirically treated with doxycycline (5 mg/kg PO q 12 h), clindamycin (10 mg/kg PO q 12 h), prednisone (1 mg/kg q 24 h) and cytosine arabinoside (100 mg/m^2^ IV CRI over 48 h) for infectious and immune mediated encephalitides. No clinical improvement was noted after 96 h of treatment with this regimen, at which time the patient was referred to VMCVM. Urine, CSF, and serum samples were submitted for canine distemper viral and *Bartonella* species PCR and cryptococcal antigen assays, all of which were negative.

**Table 1:** Serum and CSF Electrophoresis and GAD65 Antibody Titers

Sample	Total Protein (g/dl)	Albumin (%)	Globulins (%)				Anti-GAD65 Antibody Titer (IU/ml)
			α - 1	α - 2	β	γ	
Negative Control Canine Serum^a^	6.9 ± 1.0	44.7 ± 3.9	11.3 ± 2.4	14.9 ± 3.1	21.6 ± 3.5	8.5 ± 1.0	8.0 ± 1.2
SDS Canine Serum Day 1	8.1	37.1	8.2	12.2	17.8	24.7	348 ± 25.1
SDS Canine Serum Day 6	7.4	41.2	8.4	11.6	19.5	19.3	77 ± 9.5
SDS Canine Serum Day 25	7.5	NA	NA	NA	NA	NA	106 ± 17.1
SDS Canine Serum Day 100	7.8	NA	NA	NA	NA	NA	46 ± 13.1
Human AntiGAD-65 Ab Positive Serum	7.2	NA	NA	NA	NA	NA	38 ± 8.5
Control CSF^a^	0.018 ± 2.4	39.7 ± 3.5	11.3 ± 1.5	15.8 ± 1.7	20.6 ± 3.3	7.6 ± 1.2	ND
SDS Canine CSF Day 1	0.1	NA	NA	NA	NA	NA	NA
SDS Patient CSF Day 6	0.025	39.2	8.5	14.6	18.2	19.5	54 ± 8.0

*IDDM* Insulin-dependent diabetes mellitus*, A* not assayed, *ND* not detectable; all replicates below detection threshold and not different than assay blanks

^a^Control values represent means +/− SD obtained from samples obtained from 6 healthy female beagle dogs < 3 years of age

On presentation to the VMCVM (Day 5) the patient’s vital parameters were within normal limits. The dog was obese, with the remainder of the general physical examination being unremarkable. The dog demonstrated rigidity of the axial and proximal appendicular muscles resulting in lordosis (Fig. 1b) that impaired ambulation. Superimposed upon the stiffness were spontaneous and tactile stimulus-induced generalized muscular spasms that had a stereotyped appearance, characterized by an initial flexion and abduction of the limbs (Fig. 1b). Joint flexion appeared truncated due to reciprocal contraction of antagonistic muscles. This progressed to a hyperlordotic and opisthotonic posture with generalized hypertonicity (Additional file 1: Video S1) that subsided within 1 min. Episodic spasms were associated with autonomic signs including mydriasis and tachycardia. During episodes, which ranged in duration from 1–10 min, the patient appeared anxious but did not lose consciousness. The patient’s cranial nerves, postural reactions, and segmental spinal reflexes were normal between episodes. Given the results of the neurological examination, prior MRI examination and CSF analyses, differential diagnoses included a paroxysmal dyskinesia associated with generalized dystonia secondary to syringomyelia or encephalomyelitis, or other movement disorder.



**Additional file 1: Video 1.** Episodic, tactile-induced opisthotonus and generalized dystonia in a dog with SDSD and anti-GAD antibodies. (MP4 14616 kb)


Cervical vertebral radiographs were obtained to evaluate for atlanto-occipital and atlantoaxial instability and were unremarkable. Electroencephalography revealed background slowing with significant movement and muscular artifact associated with periods of hypertonicity. An EMG was also performed, and continuous motor unit activity was noted in the cervicothoracic and lumbar axial muscles at rest (Fig. 1c). Similar discharges were detectable in axial and appendicular musculature during an episode of hypertonicity. The patient was started on gabapentin (5 mg/kg PO q 12 h). The dog demonstrated variably severe but persistent muscular rigidity that was exacerbated by handling throughout the period of hospitalization, but resumed attempts to voluntarily ambulate by the end of Day 5. Baclofen (1 mg/kg PO) and levetiracetam (21 mg/kg PO q 8 h) were added to the treatment regimen.

On Day 6 general anesthesia was induced and the EMG repeated. No abnormal activity was present in axial or appendicular musculature. Cerebrospinal fluid was collected from the cerebellomedullary cistern and was grossly, cytologically, and biochemically unremarkable (WBC 1 cell/uL; RBC 0 cell/uL; protein 24.9 mg/dL); though the IgG index was elevated and anti-GAD antibodies were observed in CSF and serial serum samples obtained from the patient (Table 1). Intravenous immunoglobulin (IVIG) was administered as a CRI (1 g/kg IV over 6 h) on Day 6. On Day 7 the patient demonstrated improved ambulation, with persistent gait stiffness. There was an approximate 50% reduction in the total number of lordotic episodes per day. On Day 8 the dog was discharged from the hospital. Medications included prednisone, phenobarbital, levetiracetam, gabapentin, and baclofen.

A recheck was performed on Day 25. According to the owner, the dog had exhibited no lordotic or opisthotonic episodes during the previous 7 days. The patient was normal on general physical exam. Mild muscular hypertonicity and gait stiffness persisted, but no other abnormalities were noted, and no opisthotonic episodes were induced during the examination. An additional IVIG infusion was recommended but declined by the owner. The baclofen, prednisone, phenobarbital, levetiracetam, and gabapentin therapies were continued. The dog was rechecked by the referring veterinarian again on Day 100, at which time mild persistent gait stiffness was the only reported abnormality.

The dog was lost to follow up until 18 months later, at which time it presented to the VMCVM emergency department for joint pain and effusion, secondary to *Borrelia burgdorferi* polyarthritis. A neurological examination performed at that visit was normal. The owner reported that all previously prescribed therapies had been gradually discontinued, with the patient being off all medications for at least one year. Occasional phantom scratching was noted, but the owner reported that no episodes of lordosis or gait disturbance had been witnessed since before the Day 25 recheck.

### Glutamic acid decarboxylase (anti-GAD) antibody ELISA

Routine and electrophoretic serum and CSF analyses were performed as previously described [11]. Anti-GAD IgG (isoform 65) antibodies in serum and CSF were quantified using a human commercial ELISA kit (Euroimmun, Luebeck, Germany), performed according to the manufacturer’s instructions. Test samples consisted of 25 μl of undiluted serum or CSF, and all samples were run in triplicate. Negative controls consisted of pooled serum and CSF obtained from healthy normal beagle dogs (*n* = 6) [11]. Positive controls consisted of serum samples obtained from a human patient with insulin dependent diabetes mellitus (IDDM) that was known to have GAD65 antibodies based on western blotting and ELISA analyses. On the basis of the lowest reproducible absorbance change relative to reagent blanks, the detection limit of the assay was defined as 5.0 IU/ml. The intra- and inter-assay coefficients of variation reported by the manufacturer of this ELISA are 7.3 and 5.7%, respectively.

## Discussion

The dog described here exhibited a novel movement disorder with many similarities to human SPSD. We believe this case also reinforces the inherent difficulty that can be associated with diagnosing and classifying individual patients within the SPS spectrum. We refer to the condition reported here as SDS, although our patient did not fulfill operational criteria associated with classical SPS as defined in humans, as muscular spasms were refractory to benzodiazepine therapy, autonomic signs were observed, and the patient had concurrent syringomyelia. In addition, the clinical course of disease reported here was inconsistent with PERM or paraneoplastic SPS, as durable clinical improvement was achieved with symptomatic and immunosuppressive therapy, including IVIG, and our patient did not develop an identifiable malignancy in the 18-month follow-up period. The phenotype observed in this dog, in conjunction with the detection of a marked CSF pleocytosis and the presence of anti-GAD antibodies in CSF and serum, provide support that clinical signs in this case were associated with an autoimmune inflammatory disease. Marked CSF pleocytosis is uncommon in SPS unless associated with an underlying infection. In veterinary medicine, pleocytosis is primarily seen with auto-immune encephalitis. The prolonged remission achieved in this patient in the absence of chronic therapy for infectious or immune-mediated encephalitides would be unusual for most canine immune-mediated encephalitidies, but does completely rule out immune-mediated encephalomyelitis as a possible underlying etiology of SDS in this case.

The clinical manifestations of muscular stiffness and spasms exacerbated by tactile stimulation displayed by this dog were suggestive of spinal cord pathology that could include abnormal sensory processing or derangement or destruction of spinal cord interneuronal circuits. Although artifact was present on the EEG, the study contained enough diagnostic epochs associated with muscular spasms to confidently exclude a seizure disorder as a cause of the observed phenotype. We also consider a seizure disorder unlikely based on the relatively abrupt discontinuation of all anti-convulsant medications, with no further observed instances of abnormal activity. Although the dog did have concurrent CM and syringomyelia, which has been associated with a variety of involuntary movements, we felt the patient’s phenotype was inconsistent with segmental spinal myoclonus or propriospinal myoclonus. We did consider that the patient’s signs could represent a variant of syringomyelic dystonia, which has not been previously reported in dogs [12, 13]. CM is the most common cause of syringomyelia in dogs, but may be an incidental finding in many at risk breeds [14, 15]. However, it was thought unlikely that the CM was the predominant etiology of the patient’s phenotype since surgical treatment of the CM or syrinx was not performed, and the patient was free of dystonic episodes and off all medications 18 months after the initial diagnosis of SDSD. At the 18 month recheck examination, the owner did report that the dog displayed phantom scratching behaviors, a common manifestation of neuropathic pain associated with canine CM and syringomyelia [15]. Another possibility is that the syrinx caused or contributed to damage and loss of GABAergic spinal cord neurons, which triggered the observed immune response to the GAD antigen, which is the enzyme localized in the synaptic terminal that catalyzes the decarboxylation of glutamate to gamma aminobutyric acid (GABA).

In this patient, the observed autonomic signs, an important clinical feature of SPS, could have been from pain or anxiety associated with the episode. No specific provocative autonomic testing was undertaken so we cannot confirm the role of the primary disease state in the development of these signs. Although tetanus can cause muscular rigidity and spasms, when generalized and severe, as in the dog reported here, it is generally not episodic nor does it resolve as rapidly.

This canine patient’s phenotype, CSF pleocytosis, EMG examination, and immunological signature were similar to that reported in people with anti-GAD antibody SPS [16, 17]. High anti-GAD antibody titers were present prior to initiation of immunosuppressive therapy, and multimodal treatment resulted in a resolution of CSF inflammation, a reduction in anti-GAD antibody titer, and eventual clinical improvement. Similar to what has been reported in humans, the magnitude of the anti-GAD antibody response in this dog did not appear to correlate with the severity of clinical disease [17]. By Day 6, the patient’s serum anti-GAD titer had decreased, the previously noted CSF pleocytosis resolved, and CSF the albumin quotient normalized. However, the CSF anti-GAD IgG specific index was elevated, and the dog was still severely clinically affected. Further treatment with IVIG resulted in rapid clinical improvement. Conversely, at the Day 25 recheck examination, the serum anti-GAD titer had risen but the patient’s condition remained improved.

We conclude that dogs may spontaneously develop a movement disorder that mimics SPS in humans. Though we are unable to demonstrate causation, SDS may have an association with anti-GAD antibodies, as do 80% of human cases of SPS [5, 6]. In this case, a satisfactory clinical outcome was achieved with treatment with centrally acting muscle relaxants, anticonvulsants, and immunosuppression. Further investigations of canines with CM and syringomyelia are required to identify and phenotypically characterize possible involuntary movements associated with this disorder.
